# Innervation of human superficial fascia

**DOI:** 10.3389/fnana.2022.981426

**Published:** 2022-08-29

**Authors:** Caterina Fede, Lucia Petrelli, Carmelo Pirri, Winfried Neuhuber, Cesare Tiengo, Carlo Biz, Raffaele De Caro, Robert Schleip, Carla Stecco

**Affiliations:** ^1^Department of Neurosciences, Institute of Human Anatomy, University of Padua, Padua, Italy; ^2^Institute of Anatomy and Cell Biology, Friedrich-Alexander-University of Erlangen-Nürnberg, Erlangen, Germany; ^3^Plastic and Reconstructive Surgery Unit University of Padua, Padua, Italy; ^4^Orthopedics and Orthopedic Oncology, Department of Surgery, Oncology and Gastroenterology DiSCOG, University of Padua, Padua, Italy; ^5^Department of Sport and Health Sciences, Technical University of Munich, Munich, Germany

**Keywords:** superficial fascia, hypodermis, innervation, nerve fibers, autonomic innervation

## Abstract

The superficial fascia has only recently been recognized as a specific anatomical structure. Furthermore, whereas it is actually recognized that the innervation of the deep/muscular fascia plays a key role in proprioception and nociception, there are very few studies that have analyzed these characteristics in the superficial fascia. In this work, our group analyzed two different anatomical districts (abdomen and thigh), from volunteer patients, undergoing surgery procedures. Each sample was processed for histological analysis by Hematoxylin&Eosin, and by immunohistochemistry stainings (in 5-micron-paraffin embedded section and in cryosectioned free floating samples), with antibodies specific for nerve fibers: S100 antibody for myelinating and non-myelinating Schwann cells, PGP9.5 antibody as pan-neuronal marker, tyrosine hydroxylase for autonomic innervation. The results revealed a huge innervation: the nervous structures were found above all around blood vessels and close to adipocytes, but they penetrated also in the connective tissue itself and are found in the midst of fibro-adipose tissue. The tissue is pervaded by both thin (mean diameter of 4.8 ± 2.6 μm) and large nerve fiber bundles of greater diameter (21.1 ± 12.2 μm). The ratio S100/TH positivity was equal to 2.96, with a relative percentage of autonomic innervation with of 33.82%. In the light of these findings is evident that the superficial fasciae have a clear and distinct anatomical identity and a specific innervation, which should be considered to better understand their role in thermoregulation, exteroception and pain perception. The knowledge of the superficial fascia may improve grading and developing of different manual approach for treatments of fascial dysfunctions, and the understanding of how some factors like temperature or manual therapies can have an impact on sensitivity of the fascia.

## Introduction

The superficial fascia has only recently been recognized as a specific anatomical structure in its own right, being originally considered as included in the hypodermis. It consists of a fibrotic layer in the middle of hypodermis, dividing the superficial adipose tissue (SAT) from the deep adipose tissue (DAT), which corresponds to the membranous layer of the tela subcutanea of the Terminologia Anatomica (Abu-Hijleh et al., 2006, FIPAT). The drastic growth of articles published on the subcutaneous tissue and the superficial fascia dates back to the last 15–20 years. Furthermore, it is mainly the fields of plastic surgery and radiology that take into consideration the superficial fascia and distinguish it as a single anatomical entity: of about 2,000 articles mentioning the superficial fascia, as many as 30% refer to surgery and 10% to radiology.

Many authors emphasize the importance of an intimate knowledge of the superficial fascia system for both reconstructive and cosmetic breast surgery (Rehnke et al., 2018), and for abdominoplasty procedures (Koller and Hintringer, 2012). Song et al. (2006) demonstrated by an *ex vivo* model that the maintenance of the biomechanical properties of the superficial fascia during surgery procedures is fundamental to enhance surgical outcome through anchoring of deeper tissues. Radiologists on the other hand point out that Magnetic Resonance Imaging (MRI) is a very useful imaging technique to detect localized fascial involvement and assess its extent in trauma, infections and also neoplastic diseases (Kirchgesner et al., 2019). MRI permits also the evaluation of various forms of lymphedema, highlighting the water retention diffusely spread over the entire dermis, and an important fluid retention located in the interlobular spacing and beside the superficial fascia, together with an increase of the mean thickness of the superficial fat lobules (Idy-Peretti et al., 1998).

Until now there are very few studies that have analyzed the amount and type of innervation present in the superficial fascia. Some studies refer to the innervation of the hypodermis in general: Chi et al. (2021) demonstrated a dense sympathetic innervation in the subcutaneous fat, whereas Glatte described that post-ganglionic C-fibers of the autonomic nervous system control the thermoregulation of the subdermal plexus at the interface between hypodermis and dermis (Glatte et al., 2019). Recently, it was estimated that the entire fascial network in the human body counts approximately 250 million of nerve endings (Schleip, 2020). This leads to consider the fascia as the richest sensory tissue of the human body, drawing the attention of surgeons and clinicians.

Recently, our group (Fede et al., 2020), analyzing the various soft tissues of the human hip region, demonstrated that the superficial fascia was the second most highly innervated tissue (with a mean density of nerves of 33.0 ± 2.5/cm^2^) after the skin (64.0 ± 5.2/cm^2^). Also SAT (superficial adipose tissue) and DAT (deep adipose tissue) were less innervated with respect to the fascia, with a mean density of innervation equal to 14.5 ± 1.6/cm^2^ and 15.0 ± 6.3/cm^2^, respectively. That work, however, was only a quantitative analysis performed by a general marker (S100 antibody), that does not allow to distinguish the autonomic or sensory innervation.

Thus, in the present work, the innervation of the human superficial fascia of the abdomen and of the hip region were studied to identify the presence of neural structures and their distribution, with a particular focus to the autonomic nervous structures. An improved knowledge of the fascial tissue innervation may lead to better understanding of the sensitivity of the superficial fascia, and may help to demonstrate how some factors like temperature or manual therapies can have an impact on some dysfunctions related to the superficial fascia.

## Materials and Methods

### Samples collection

The Superficial Fascia (SF, 700 μm thickness, Pirri et al., 2022) was collected in the abdominal region (from three volunteer patients who were undergoing abdominoplasty at the Plastic and Reconstructive Surgery Unit of the University of Padua), and in the hip area (from six volunteer patients undergoing elective surgical procedures at the Orthopaedic Clinic of the University of Padua).

The ethical regulations regarding research on human tissues were carefully followed (approval no. 3722/AO/16, study approved on 21 April 2016 by the Ethical Committee for clinical trials in the province of Padova). The research was performed in accordance with the ethical standards of the 1964 Declaration of Helsinki as revised in 2000 and those of Good Clinical Practice. All subjects participating in the study received a thorough explanation of the risks and benefits of inclusion and gave their oral and written informed consent to publish the data.

All the samples were at least 0.5 cm × 1.5 cm, and derived from adults: two females/one male, age between 37 and 45, in the hip area, and three females/three males, age >55, in the abdomen region.

Samples of superficial fascia from the hip region were all formalin-fixed (in 10% buffered formaldehyde, pH 7.4) for immunohistochemistry analysis and histological evaluation.

In the abdominal region, given the large area where the samples are taken, 8–10 specimens per subject were taken in different areas of the SF, in about 40 cm^2^ of total area, in full thickness (from skin to SF). One specimen per subject was evaluated in full thickness by hematoxylin and eosin stain, in the others the SF was isolated and cleansed of superficial and deep adipose tissue.

The isolated SF from abdomen, fixed in 10% buffered formaldehyde, pH 7.4, were divided in two groups: three SF (for each patient) were used for floating immunohistochemistry, the other 5–7 SF followed the protocol for classical paraffin embedding.

For floating immunohistochemistry, the SF samples were washed in Phosphate Buffered Saline (PBS) and transferred in water with increasing series of cryo-preservative solution (sucrose 10%–20%–30% w/v, 5 days for each solution), and then were frozen in Isopentane (2-Methylbutane-Merck) cooled at −80°C in ice dry. Sections of 60 μm were cut at −20°C in a Cryostat (Leica—CM1850), then washed in PBS and processed for floating Immunohistochemistry (see paragraph below).

**Figure 1:**
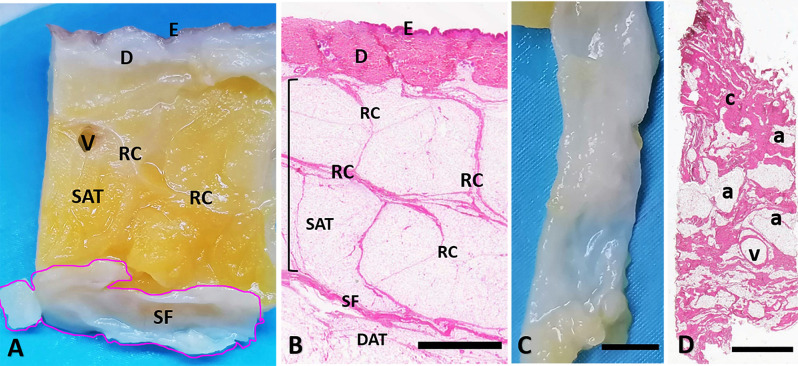
Superficial fascia layer of the abdomen. **(A)** Formalin-fixed sample of the abdominal region, from skin (E: epidermis, D: dermis) to the superficial fascia (SF). **(B)** Hematoxylin and eosin stain of all the layers from skin (E: epidermis, D: dermis), to the deep adipose tissue (DAT). The superficial fascia (SF) is the fibrous layer localized between the superficial adipose tissue (SAT, organized in lobules separated by the fibrous septa of the retinacula cutis, RC) and the deep adipose tissue (DAT). The SF highlighted in **(A)** is isolated from the SAT and the DAT **(C)** and stained by Hematoxylin and Eosin **(D)**. Panel **(D)** is a tangential section of the flat embedded SF as in **(C)**. Histologically the SF layer if formed by a net of collagen fibers arranged irregularly (c: connective tissue), interconnected and mixed with adipocytes (a) and crossed by blood vessels (v). Scale Bars = 3 mm.

The other fixed samples were dehydrated in graded ethanol and in xylene, and embedded in paraffin. Five-μm tangential sections were cut by microtome, dewaxed and hydrated for Hematoxylin and Eosin stain (Figure 1D), to evaluate the general morphology and organization of the tissue, and for Immunohistochemical protocol, as detailed in the paragraph below.

### Immunohistochemistry

Dewaxed sections and floating samples were treated with blocking of endogenous peroxidases (1% H_2_O_2_ in PBS), incubated in blocking solution [PBS + 0.2% bovine serum albumin (BSA)] for 1 h and then incubated overnight at 4°C with Rabbit Polyclonal Anti S100 (marker for myelinating and non-myelinating Schwann cells, Agilent-Dako, dilution 1:4,000), Rabbit Anti Tyrosine Hydroxylase (TH, for autonomic innervation, GeneTex, dilution 1:500), or Rabbit Anti PGP9.5 (as a pan-neuronal marker, Merck Millipore, dilution 1:500). After repeated PBS washing, the samples were incubated with the secondary Goat anti rabbit (Jackson, dilution 1:300) for 1 h. Negative controls were processed with the same protocol with the omission of the primary antibodies. The reaction was then developed with 3,3’-diaminobenzidine (Liquid DAB + substrate Chromogen System kit Dako) and stopped with distilled water. Samples were finally counterstained with hematoxylin.

The images were acquired by using Leica DMR microscope (Leica Microsystems, Wetzlar, Germany).

### Image analysis

The image analysis was performed only in the samples collected from abdominoplasty, given the large area and the higher precision of collection, which can permit a more accurate analysis. Images were acquired through Leica DMR (Leica Microsystems, Wetzlar, Germany) and computerized image analysis was performed with ImageJ software in serial sections (at least 20 pictures for each sample, enlargement 10×), to quantify the area (%) positive to Tyrosine Hydoxylase and S100 and to calculate the S100/TH ratio.

In addition, the mean diameter of the nerve fiber bundles was calculated by ImageJ software (enlargement 40×) in the three samples from abdomen, in at least 15 S100-positive images per sample. Recognitions of axons by light microscopy can overestimate the diameter of the nervous fibers, for the possible inaccurate identification of very small fibers: our analysis permitted however the identification of bundles of axons up to 2 μm of diameter.

## Results

**Figure 2:**
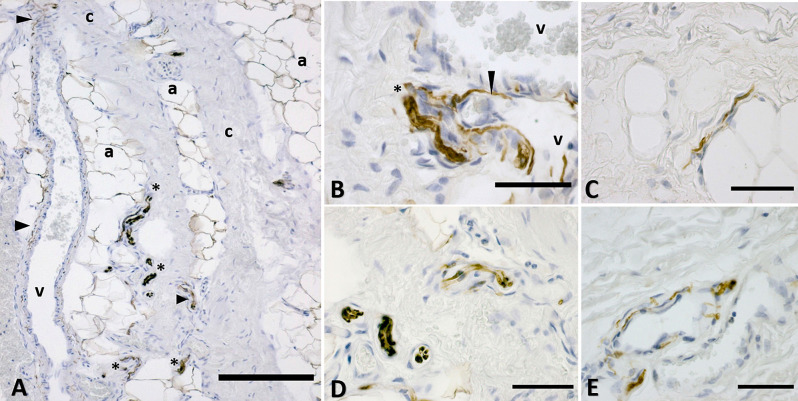
Distribution of the innervation in superficial fascia: immunohistochemistry by S100 antibody. **(A)** The connective tissue (c) is crossed by blood vessels (v) and interconnected with areas rich in adipocytes (a). Nervous structures (*indicates big nerves, arrowheads indicate thin nerve fibers) are evident between the collagen fibers, near the adipocytes and in the wall of blood vessels. **(B)** A nerve (*) and thin nerve fibers (arrow) close to two blood vessels (v). **(C)** Innervation between the adipocytes area. **(D)** Nerves crossing the fibrous connective tissue of the SF. **(E)** Innervation of the wall of a blood vessel supplying the SF. a, adipocyte; v, vessel; c, connective tissue; *, nerve. Arrowheads indicate small nerve fibers. Scale Bars: **(A)** = 200 μm, **(B–E)** = 50 μm.

The full thickness specimen (from skin to SF, Figures 1A,B) highlighted as superficial fascia (SF) divides the superficial adipose tissue (SAT), organized in fat lobules with evident fibrous septa (retinacula cutis superficialis), from the deep adipose tissue (DAT). SF is a well-defined thin fibrous layer (Figure 1C), approximately 500–700 μm thick. The Hematoxylin and Eosin stain (Figure 1D) of the SF demonstrated as it is formed by fibro-fatty connective tissue: the collagen fibers are irregularly arranged, spatially interconnected and mixed with adipocytes, and pervaded by blood vessels of different caliber supplying and crossing the tissue. The same organization was evident also in Figure 2A: collagen fibers form a net, between areas of adipocytes and large vessels. The immunohistochemistry by S100 antibody revealed the presence of a huge innervation in the superficial fascia, above all in the wall of the blood vessels (Figures 2A,B,E) and near the adipocytes (Figures 2A,C), but also in the connective tissue itself (Figures 2A,D). The nervous structures pervading the tissue are both thin nerve fiber bundles (mean diameter of 4.8 ± 2.6 μm) and larger nerve fiber bundles of bigger diameter (21.1 ± 12.2 μm; Figure 2D), with approximately the same distribution.

**Figure 3:**
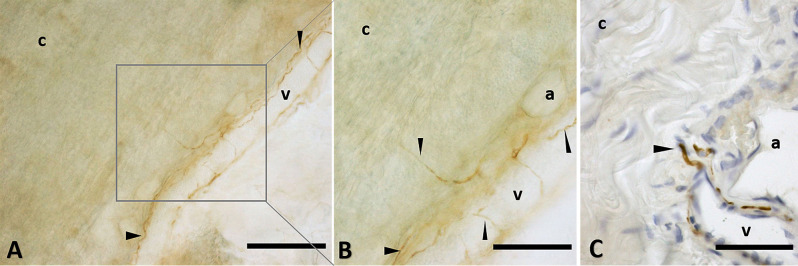
Nerve fibers penetrating the connective tissue. Innervation in a free floating superficial fascia of the abdomen region, by anti-S100 antibody. **(A)** The wall of the blood vessel (v) is richly innervated, and some nerve fibers enter inside the connective tissue (c) of the SF, as shown in the box, enlarged in **(B)**. Panel **(C)** shows the same anti-S100 reaction in paraffin-embedded 5 μm section. The arrows indicate the nerve fibers. v, blood vessel; c, connective tissue; a, adipocyte. Scale bars: **(A)** = 200 μm; **(B)** = 100 μm; **(C)** = 50 μm.

**Figure 4:**
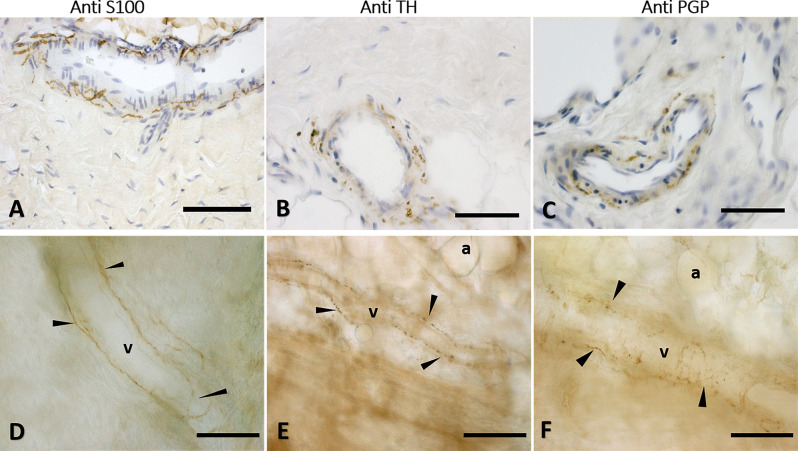
Innervation of the blood vessels of superficial fascia. Superficial fascia of the abdomen, stained with S100 **(A,D)**, Tyrosine Hydroxylase **(B,E)** and PGP9.5 **(C,F)** antibodies. Panels **(A,B,C)** are paraffin-embedded-5 μm samples, whereas **(D,E,F)** are free-floating samples. All the pictures show the innervation of blood vessels. In **(E,F)** the varicose nature of the axons is evident. v, blood vessel; a, adipocyte; arrows indicate the nerve fibers. Scale bars: **(A,F)** = 100 μm; **(B,C)** = 50 μm; **(D,E)** = 200 μm.

**Figure 5:**
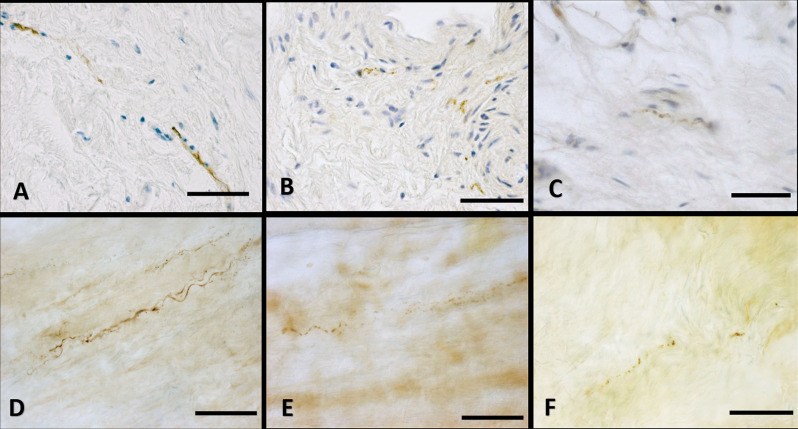
Innervation of the connective tissue. Superficial fascia of the hip (paraffin-embedded-5 μm section: **A,B,C**) and of abdomen (free-floating samples: **D,E,F**) stained with S100 **(A,D**, respectively), Tyrosine Hydroxylase **(B,E)** and PGP 9.5 **(C,F)** antibodies. Scale bars: **(A,D,E)** = 100 μm; **(B,C,F)** = 50 μm.

**Figure 6:**
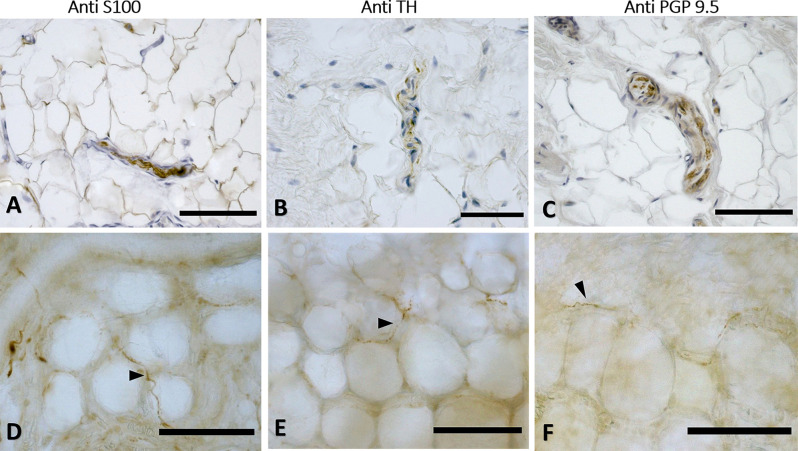
Innervation in the adipocyte areas: immunohistochemistry with anti-S100, anti-Tyrosine Hydroxylase, and anti-PGP9.5 antibodies. Superficial fascia of the hip (paraffin-embedded-5 μm section: **A,B,C**) and of abdomen (free-floating sample: **D,E,F**) stained with S100 **(A,D)**, Tyrosine Hydroxylase **(B,E)** and PGP9.5 **(C,F)** antibodies. Arrowheads indicate single nerve fibers. Scale bars: **(A,C–F)** = 100 μm, **(B)** = 50 μm.

**Figure 7:**
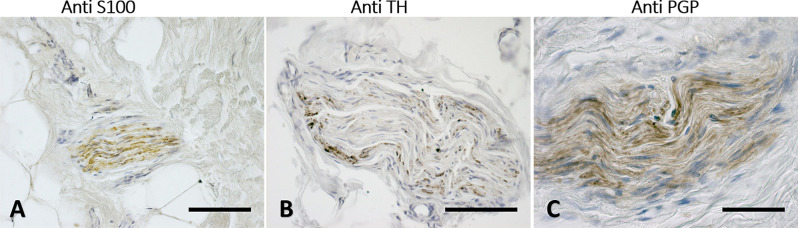
Nerves fascicles passing through SF. Superficial fascia of the hip **(A)** and of the abdomen **(B,C)** showing thick fascicles in the SF tissue, stained with S100 **(A)**, Tyrosine Hydroxylase **(B)**, and PGP9.5 **(C)** antibodies. Scale bars: **(A,C)** = 100 μm; **(B)** = 200 μm.

The immunohistochemistry analyses, both in paraffine-embedded sections and in free-floating samples, permitted to demonstrate a rich innervation of the superficial fascia. The nerves are particularly concentrated in the blood vessels’ adventitia (Figures 3, 4), but they penetrate also in the connective tissue without obvious vascular relation (Figure 3) and are found in the midst of the fibro-adipose tissue (Figures 5, 6). The innervation can be considered both sensory and autonomic (sympathetic), because nerve fibers reactive for S100 and PGP 9.5, a pan-neuronal marker, outnumber those reactive for TH (Figures 3, 4, 5, and 6). In general, the reaction with PGP 9.5 antibody showed thinner positive fibers less contrasted around the blood vessels (Figures 4C,F) and in the dense connective areas (Figures 5C,F), with some varicosities in the axons. At the same time, there is a huge innervation also in the looser connective areas: this is characterized by wavy collagen fibers, large blood vessels that branch out into the dense tissue, nerves and groups of adipocytes. In that areas some large nerve fascicles (up to 200 μm of diameter) cross the SF, presumably on their way to the skin, and showed positive reactions to all the markers: S100 (Figure 7A), TH (Figure 7B), PGP 9.5 (Figure 7C). TH positive nerve fibers were outnumbered by those immunostained by the general markers S100 and PGP 9.5.

**Figure 8:**
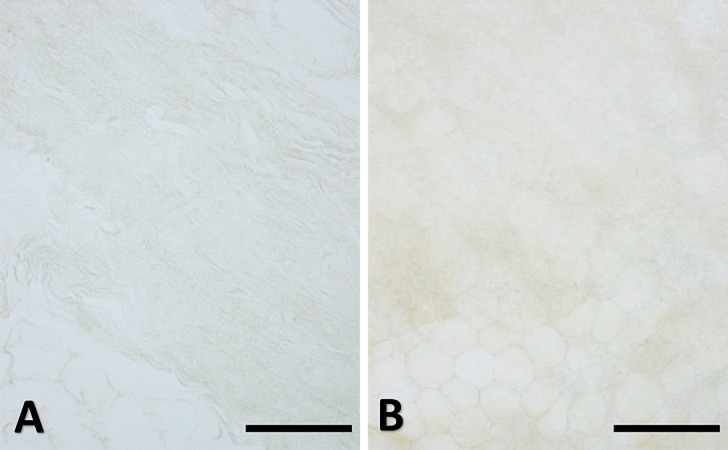
Negative controls. Superficial fascia of the hip (**A**, paraffin-embedded-5 μm section) and of the abdomen (**B**, free-floating sample) with the omission of the primary antibody: the negative reaction confirmed the specificity of the immuno-reactions. Scale bars: 100 μm.

No corpuscular sensory structures (like Pacini or Ruffini) were found in the SF samples, nether in the hip region nor in the abdomen. The specificity of the immunostaining was demonstrated by the absence of reaction in the negative controls (Figure 8).

The fraction of area (%) positive to Tyrosine Hydoxylase and S100 was estimated to calculate the S100/TH ratio. The analysis was performed only in the abdomen area given the higher quality of the tissue samples from that area and the consequently more reliable analysis. Given the higher background of PGP 9.5 antibody and the less contrasted reaction, the ratio calculation was performed on S100-stained images and TH-stained images, to have a relative quantification of the autonomic innervation in the SF. The immunohistochemistry showed that the TH positive area (IR area %) is around 0.326%, whereas the S100 positive area is around 0.964%, thus leading to a ratio S100/TH positivity of 2.96.

## Discussion

This work is the first histological investigation focused on the human SF innervation, which demonstrates that it is hugely innervated, both with autonomic and sensory nerve fibers, according also to our previous work demonstrating that SF of the hip region was the most highly innervated tissue after the skin (skin>Sup Fascia>Deep Fascia>DAT>SAT; Fede et al., 2020). Only some large nerve fascicles cross the superficial fascia to reach the skin, the majority of nerve fibers are located around the vessels, innervating their wall, or are embedded in the fibro-adipose tissue. The perivascular innervation displays a plexus structure in the SF. The floating immunohistochemistry was very helpful to highlight the nervous fibers 3D network and distribution: they are concentrated above all around the vessels, but also in the areas of looser connective tissue, characterized by wavy collagen fibers, large vessels and groups of adipocytes. Furthermore, in the dense connective areas of SF some vessels were noted: it is likely that they are branches of larger vessels located in loose connective areas. The nervous structures in the dense connective tissue of SF can be divided in two groups: thin nerve fiber bundles of 4.8 ± 2.6 μm diameter, and bigger nerve fascicles with a mean diameter of 21.1 ± 12.2 μm. These data are in accord with the values reported in the human hip joint area, in which the SF showed thin nervous structure of 19.1 ± 7.2 μm, with respect to large nerve bundles crossing the muscle with a diameter of 36.4 ± 13.4 μm (Fede et al., 2020). In this work, the only big nerves observed were located in the looser connective tissue areas, constituting passage nerve structures, compared to the small nerve fibers found in majority in the dense tissue. Unexpectedly, no Pacini corpuscles, which mediate vibration and pressure sensations, and Ruffini corpuscles, which perceive stretching, were found in these fasciae, also if they are usually described in the hypodermis (Cobo et al., 2021). The specific innervation of the superficial fascia and its strong relationship with the skin suggest that this innervation is part of the dermatomeric perception (Stecco et al., 2019).

In this study it was not possible to make a quantification and a comparison between the two investigated regions (abdomen and hip), for the low number of collected samples. However, the percentage of S-100 positive area calculated in the abdomen was 0.964% ± 0.110%, showing a bigger innervation with respect to the innervation of the hip area revealed by our group in a precedent article, equal to 0.22% ± 0.06% (Fede et al., 2020). The huge innervation by thin nerve fibers in the SF and the presence of single fibers in the middle of collagen bundles confirm the sensory role of SF and its possible implications in nociception. These nervous structures were positive to S100, PGP 9.5, and TH.

The positivity to TH-staining confirmed the presence of a sympathetic autonomic nervous system inside the fascia, suggesting the possible role of the autonomic nervous system in the regulation of vascularization of the superficial fascia, but probably also of the more superficial layers, as the skin (Sheng and Zhu, 2018). The relative percentage of autonomic innervation in SF samples was equal to 33.82%, not so distant to the amount reported by Mense, who affirmed that approximately 40% of the entire fascia innervation consisted of postganglionic sympathetic fibers (Mense, 2019). Probably, the majority of these fibers are vasoconstrictors; however, some of the sympathetic endings seem to serve an unknown function as they do not terminate on the vessels, as demonstrated also in this study. This was reported also by a recent work by Neuhuber and Jänig, who demonstrated the presence of Aδ and C fibers and autonomic nervous fibers in thoracolumbar fascia of rats not associated with blood vessels, but inside the connective tissue with a probable trophic activity (Neuhuber and Jänig, 2017). A recent work (Larsson and Nagi, 2022) highlighted the role in the bidirectional pain modulation of some unmyelinated C-fibers, termed C low-threshold mechanoreceptors (C-LTMRs), positive for TH staining, both in human and animal studies, especially in the skin. A role of some TH-positive nervous fibers in the connective tissue of SF as pain modulator mechanoreceptors, which allow to mediate mechanical allodynia evoked by touch in conditions such as inflammatory pain, cannot be excluded. The sympathetic activity may have also a role in the control of fascial tone: Staubesand and Li (1996) proposed a close connection between fascial stiffness and sympathetic activation. By this way, the chronic stress can affect the basal tone of the fascial tissue, by the activation of the autonomic nervous system: recent works demonstrated, in fact, the influence of the sympathetic nervous system on the TGF-β1 expression, which in turn stimulates myofibroblast contractility (Bhowmick et al., 2009; Schleip et al., 2019). So, the presence of TH-positive fibers helps to state that a condition of stress, a trauma, or a sudden change in temperature may increase the sympathetic activity not only in the skin, but also in the superficial fascia (Scheff and Saloman, 2021; Ishikawa and Furuyashiki, 2022). This can help to understand how a state of chronic stress may create a change of the superficial fascia, causing some alterations in thermoregulation, lymphatic flow and venous circulation. The changes caused by the chronic stress can also influence the immune system: the TGF-β1/Smad2/3/Foxp3 axis was remarkably activated following chronic stress, causing lymphocyte apoptosis and immunosuppression (Zhang et al., 2018). These aspects can suggest a possible involvement of the SF in the mechanism of fibromyalgia: Evdokimov et al. (2020) demonstrated that in patients affected by fibromyalgia the dermal nerve fiber length of fibers with vessel contact was reduced, suggesting a possible relationship between sympathetic neurons and impaired thermal tolerance commonly reported by fibromyalgic patients.

Also the presence of a scar in the skin can influence an alteration of the SF: scars are in fact more severe and painful when the subcutaneous fascia beneath the dermis is injured upon surgical or traumatic wounding. A recent ultrasound imaging study revealed a thickening of the subcutaneous tissue of an injured knee in a young female patient (right/injured= 2.33 mm, left = 1.31 mm) and, in particular, of the SF with hyperechoic thickening of the retinacula cutis, causing significant pain (Pirri et al., 2020). It was demonstrated that the fascial tissue contains specialized sentry fibroblasts, which collectively migrate after injury, progressively causing a contraction of the skin and scar formation (Correa-Gallegos et al., 2019; Jiang et al., 2020). It cannot be excluded that the autonomic innervation may have a role in this process of regulation of scar formation.

Finally, the huge innervation of the SF can help to assume that a manual light superficial massage can have effects on the autonomic nervous system, helping to improve the symptoms related to dysfunction of the superficial venous system, or thermo-regulation. In conclusion, we can affirm that the SF has a clear and distinct anatomical entity and a specific innervation, which should be considered to improve grading and developing of different manual approach for treatments of fascial dysfunctions, and the understanding of how some factors like temperature or manual therapies can have an impact on nociception and sensitivity of the fascia.

## Limitations and Further Research

Several limitations of this work should be declared. First, our analysis was based on only a small population sample: further studies with larger sample sizes from various anatomical regions are needed to establish the reliability and validity of our findings. Through a systematic study of the innervation of the SF, it will be possible to highlight some hypothetical variations due to the topographic region. Furthermore, it will be useful to broaden the evaluation of the innervation also to SAT and DAT in the same experimental conditions, to visualize possible variations with respect to the SF, such as the presence of Pacini and Ruffini corpuscles. It should also be added that by this work we did not use specific markers (anti-SP, CGRP or NGF), to demonstrate the presence of neuropeptides in the varicosities of the axons, so we have not demonstrated with certainty the presence of nerve terminals in the SF. Anyway, the small diameter of the nerve fibers inside the tissue and the aspects with varicosities, as indicated in the literature (Mense, 2019) and as visible by PGP9.5 staining (Figures 4C,F, 5C,F), lead to affirm that these are not passing fibers but a specific innervation of the superficial fascia. In the next future, a systematic and specific study will allow to identify the presence of nerve endings. Lastly, further studies are necessary to analyze the presence of nociceptive fibers in the SF, to better understand the role of this tissue in pain perception and consequently to better target physical therapies.

## Data Availability Statement

The original contributions presented in the study are included in the article, further inquiries can be directed to the corresponding author.

## Ethics Statement

The studies involving human participants were reviewed and approved by Ethical Committee for clinical trials in the province of Padua. The patients/participants provided their written informed consent to participate in this study.

## Author Contributions

CF: manuscript drafting, image analysis, and immunohistochemistry. LP: responsible for samples fixation, embedding, and for immunostainings. CP: participation in manuscript writing, image analysis, and statistical analyisis. WN and RS: participation in immunohistochemistry coordination and manuscript writing and editing. CT and CB: fascia collecting from the volunteers. RDC and CS: participation in manuscript writing and editing, and in work coordination. All authors contributed to the article and approved the submitted version.

## Funding

This research work was supported by Verein zur Förderung der Faszienforschung e.V. (Fascia Research Charity Assoc.).

## Conflict of Interest

The authors declare that the research was conducted in the absence of any commercial or financial relationships that could be construed as a potential conflict of interest.

## Publisher’s Note

All claims expressed in this article are solely those of the authors and do not necessarily represent those of their affiliated organizations, or those of the publisher, the editors and the reviewers. Any product that may be evaluated in this article, or claim that may be made by its manufacturer, is not guaranteed or endorsed by the publisher.

## References

[B1] Abu-Hijleh M. F. Roshier A. L. Al-Shboul Q. Dharap A. S. Harris P. F. (2006). The membranous layer of superficial fascia: evidence for its widespread distribution in the body. Surg. Radiol. Anat. 28, 606–619. 10.1007/s00276-006-0142-8 17061033

[B2] Bhowmick S. Singh A. Flavell R. A. Clark R. B. O’Rourke J. Cone R. E. (2009). The sympathetic nervous system modulates CD4^+^FoxP3^+^ regulatory T cells via a TGF-β-dependent mechanism. J. Leukoc. Biol. 86, 1275–1283. 10.1189/jlb.0209107 19741161 PMC2780915

[B4] Chi J. Lin Z. Barr W. Crane A. Zhu X. G. Cohen P. (2021). Early postnatal interactions between beige adipocytes and sympathetic neurites regulate innervation of subcutaneous fat. eLife 10:e64693. 10.7554/eLife.64693 33591269 PMC7990502

[B5] Cobo R. García-Piqueras J. Cobo J. Vega J. A. (2021). The human cutaneous sensory corpuscles: an update. J. Clin. Med. 10:227. 10.3390/jcm10020227 33435193 PMC7827880

[B6] Correa-Gallegos D. Jiang D. Christ S. Ramesh P. Ye H. Wannemacher J.. (2019). Patch repair of deep wounds by mobilized fascia. Nature 576, 287–292. 10.1038/s41586-019-1794-y 31776510

[B9] Evdokimov D. Dinkel P. Frank J. Sommer C. Üçeyler N. (2020). Characterization of dermal skin innervation in fibromyalgia syndrome. PLoS One 15:e0227674. 10.1371/journal.pone.0227674 31929578 PMC6957156

[B11] Fede C. Porzionato A. Petrelli L. Fan C. Pirri C. Biz C.. (2020). Fascia and soft tissues innervation in the human hip and their possible role in post-surgical pain. J. Orthop. Res. 38, 1646–1654. 10.1002/jor.24665 32181900

[B12] FIPAT (2011). Terminologia Anatomica: International Anatomical Terminology. New York: Thieme.

[B13] Glatte P. Buchmann S. J. Hijazi M. M. Illigens B. M. Siepmann T. (2019). Architecture of the cutaneous autonomic nervous system. Front. Neurol. 10:970. 10.3389/fneur.2019.00970 31551921 PMC6746903

[B14] Idy-Peretti I. Bittoun J. Alliot F. A. Richard S. B. Querleux B. G. Cluzan R. V. (1998). Lymphedematous skin and subcutis: *in vivo* high resolution magnetic resonance imaging evaluation. J. Invest. Dermatol. 110, 782–787. 10.1046/j.1523-1747.1998.00184.x 9579546

[B15] Ishikawa Y. Furuyashiki T. (2022). The impact of stress on immune systems and its relevance to mental illness. Neurosci. Res. 175, 16–24. 10.1016/j.neures.2021.09.005 34606943

[B16] Jiang D. Christ S. Correa-Gallegos D. Ramesh P. Kalgudde Gopal S. Wannemacher J.. (2020). Injury triggers fascia fibroblast collective cell migration to drive scar formation through N-cadherin. Nat. Commun. 11:5653. 10.1038/s41467-020-19425-1 33159076 PMC7648088

[B17] Kirchgesner T. Tamigneaux C. Acid S. Perlepe V. Lecouvet F. Malghem J.. (2019). Fasciae of the musculoskeletal system: MRI findings in trauma, infection and neoplastic diseases. Insights Imaging 10:47. 10.1186/s13244-019-0735-5 31001705 PMC6473016

[B18] Koller M. Hintringer T. (2012). Circumferential superficial fascia lift of the lower trunk: surgical technique and retrospective review of 50 cases. J. Plast. Reconstr. Aesthet. Surg. 65, 433–437. 10.1016/j.bjps.2011.09.045 22062973

[B20] Larsson M. Nagi S. S. (2022). Role of C-tactile fibers in pain modulation: animal and human perspectives. Curr. Opin. Behav. Sci. 43, 138–144. 10.1016/j.cobeha.2021.09.005

[B23] Mense S. (2019). Innervation of the thoracolumbar fascia. Eur. J. Transl. Myol. 29:8297. 10.4081/ejtm.2019.8297 31579474 PMC6767935

[B24] Neuhuber W. Jänig W. (2017). Fascia in the Osteopathic Field, eds Liem T. Tozzi P. Chila A. Scotland: Handspring Publishing Limited.

[B25] Pirri C. Fede C. Petrelli L. Guidolin D. Fan C. De Caro R.. (2022). Elastic fibres in the subcutaneous tissue: is there a difference between superficial and muscular fascia? A cadaver study. Skin Res. Technol. 28, 21–27. 10.1111/srt.13084 34420234 PMC9907621

[B26] Pirri C. Stecco A. Fede C. De Caro R. Stecco C. Özçakar L. (2020). Ultrasound imaging of a scar on the knee: sonopalpation for fascia and subcutaneous tissues. Eur. J. Transl. Myol. 30:8909. 10.4081/ejtm.2019.8909 32499900 PMC7254433

[B27] Rehnke R. D. Groening R. M. Van Buskirk E. R. Clarke J. M. (2018). Anatomy of the superficial fascia system of the breast: a comprehensive theory of breast fascial anatomy. Plast. Reconstr. Surg. 142, 1135–1144. 10.1097/PRS.0000000000004948 30511967 PMC6211786

[B28] Scheff N. N. Saloman J. L. (2021). Neuroimmunology of cancer and associated symptomology. Immunol. Cell. Biol. 99, 949–961. 10.1111/imcb.12496 34355434 PMC9250294

[B29] Schleip R. (2020). “Innervation of Fascia,” in Fascia, Function and Medical Applications, eds Lesondak D. Akey A. M. (United Kingdom: Taylor & Francis Ltd), 61–69. 10.1201/9780429203350-5

[B30] Schleip R. Gabbiani G. Wilke J. Naylor I. Hinz B. Zorn A.. (2019). Fascia is able to actively contract and may thereby influence musculoskeletal dynamics: a histochemical and mechanographic investigation. Front. Physiol. 10:336. 10.3389/fphys.2019.00336 31001134 PMC6455047

[B31] Sheng Y. Zhu L. (2018). The crosstalk between autonomic nervous system and blood vessels. Int. J. Physiol. Pathophysiol. Pharmacol. 10, 17–28. 29593847 PMC5871626

[B32] Song A. Y. Askari M. Azemi E. Alber S. Hurwitz D. J. Marra K. G.. (2006). Biomechanical properties of the superficial fascial system. Aesthet. Surg. J. 26, 395–403. 10.1016/j.asj.2006.05.005 19338921

[B33] Staubesand J. Li Y. (1996). Zum Feinbau der Fascia cruris mit besonderer Beruücksichtigung epi- und intrafaszialer Nerven. Manuelle Medizin 34, 196–200.

[B35] Stecco C. Pirri C. Fede C. Fan C. Giordani F. Stecco L.. (2019). Dermatome and fasciatome. Clin. Anat. 32, 896–902. 10.1002/ca.23408 31087420

[B38] Zhang H. Caudle Y. Wheeler C. Zhou Y. Stuart C. Yao B.. (2018). TGF-β1/Smad2/3/Foxp3 signaling is required for chronic stress-induced immune suppression. J. Neuroimmunol. 314, 30–41. 10.1016/j.jneuroim.2017.11.005 29169800 PMC5756116

